# Bacterial Porins and Their Procoagulant Role: Implication in the Pathophysiology of Several Thrombotic Complications during Sepsis

**DOI:** 10.3390/toxins16080368

**Published:** 2024-08-20

**Authors:** Carmine Siniscalchi, Alessandro Perrella, Ugo Trama, Francesca Futura Bernardi, Egidio Imbalzano, Giuseppe Camporese, Vincenzo Russo, Olga Scudiero, Tiziana Meschi, Pierpaolo Di Micco

**Affiliations:** 1 Internal Medicine Unit, Department of Internal Medicine, University of Parma, 43100 Parma, Italy; csiniscalchi84@gmail.com (C.S.); tiziana.meschi@unipr.it (T.M.); 2 UOC Emerging Infectious Disease and High Contagiousness, AORN dei Colli, P.O. Cotugno, 80131 Naples, Italy; alessandro.perrella@ospedaledeicolli.it; 3 UOD Politica del Farmaco e Dispositivi della DG per la Tutela della Salute SSR, Regione Campania, Via Santa Lucia, 80100 Napoli, Italy; ugo.trama@regione.campania.it; 4 Department of Pharmacology, University of Campania “Luigi Vanvitelli”—Monaldi Hospital, Piazzale Ettore Ruggeri, 80131 Naples, Italy; francescafutura.bernardi@regione.campania.it; 5 Department of Clinical and Experimental Medicine, University of Messina, 98100 Messina, Italy; 6 Department of Internal Medicine DIMED, Padua University Hospital, 35100 Padua, Italy; giuseppe.camporese@aopd.veneto.it; 7 Cardiology Unit, Department of Translational Medical Sciences, University of Campania “Luigi Vanvitelli”—Monaldi Hospital, Piazzale Ettore Ruggeri, 80131 Naples, Italy; vincenzo.russo@unicampania.it; 8 Department of Molecular Medicine and Medical Biotechnology, University Federico II of Naples, 80131 Naples, Italy; olga.scudiero@unina.it; 9 UOC Medicina Interna, AFO Medica, P.O. Santa Maria delle Grazie, ASL Napoli 2 Nord, Pozzuoli, 80078 Naples, Italy; pdimicco@libero.it

**Keywords:** porins, thromboembolism, bacteria, sepsis

## Abstract

The association between sepsis and thrombotic complications is still not well known. Different mechanisms have been shown to be involved in the sepsis-induced prothrombotic state, but clinical scenarios may differ. In this review, we have summarized the role that bacterial products such as porins and toxins can have in the induction of the prothrombotic state during sepsis and the interaction that they can have with each other. Furthermore, the above-mentioned mechanisms might be involved in the pattern of the clinical presentation of thrombotic events during bacterial sepsis, which would secondarily explain the association between sepsis and venous thromboembolism, the association between sepsis and disseminated intravascular coagulation, and the association between sepsis and microangiopathic venous thromboembolism.

## 1. Introduction

Thrombotic complications have been frequently reported during infections, and Gram-negative bacteria are more commonly associated with these kinds of complications. Several pathophysiological mechanisms have been reported as the causes of a septic-associated hypercoagulable state, and contemporaneous mechanisms may be induced by a single infection. Further thrombotic risk factors for thrombosis may also be present in the host at the time of infection. Infection itself is considered one of the major prothrombotic risk factors required for pharmacological thromboprophylaxis according to the PADUA score.

**Figure 1:**
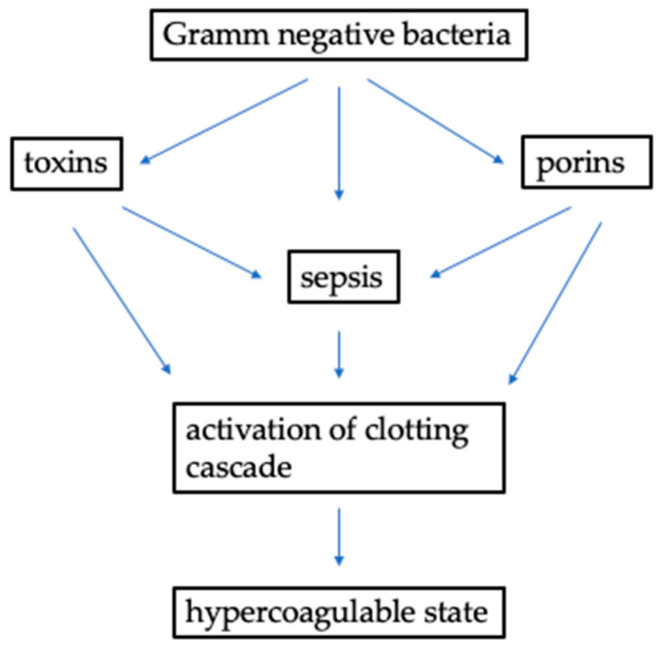
Bacterial infection and hypercoagulable state.

Procoagulant actions exerted by bacteria have been identified in vitro and in vivo. In vitro tests, in fact, have already divided bacteria into two subgroups based on their ability to produce an enzyme that differentiates them in coagulase-positive and coagulase-negative bacteria. This difference also seems to identify more aggressive subspecies of bacteria in vivo (see Figure 1).

Yet, in vivo research has underlined several conditions induced by the presence of infections that are able to induce a hypercoagulable state. First of all, bacterial toxins are specific products of bacteria capable of activating a clotting cascade with an induced infective hypercoagulable state. Other bacterial products such as porins have also been identified as a prothrombotic mechanism present in patients with septic manifestations.

Here, we describe the main mechanisms exerted by bacteria in the pathophysiology of thrombotic complications in vivo such as their association with infection and venous thromboembolism (VTE), with disseminated intravascular coagulation (DIC), and with thrombotic microangiopathies (TAM). Table 1 shows the most frequent bacteria involved in sepsis and their characteristics.

## 2. Sepsis-Induced Coagulopathy

Sepsis-induced coagulopathy (SIC) is a major complication in sepsis. SIC is associated with an increase in mortality and is present in about 20% of patients with sepsis [1,2]. The principal characteristics of SIC are coagulation activation, leading to microvascular thrombi, systemic inflammation, and impaired organ perfusion with organ dysfunction. The innate immune and coagulation systems strongly interact during SIC. Pathogen-associated molecular patterns (PAMPs) are fundamental in SIC. PAMPs are molecular fragments of membrane components, glycoproteins, and nucleic acids of pathogens. Patterns of these molecules are recognized by host receptors, including Toll-like receptors (TLRs) [3], and they trigger the activation of both cellular and noncellular components of the innate immune system [4]. Bacterial cell membrane components are well-recognized PAMPs and are linked to procoagulant activity. In addition, the proinflammatory cytokines released during septicemia are involved in the prothrombotic state. Proinflammatory cytokines such as IL1, IL2, IL4, IL6, IL8, IL10, TNF alpha, IFN gamma, and C5a may negatively impact the procoagulant state. In particular, cytokine storms during sepsis, such as interleukin (IL)1-alpha in LPS-induced Caspase 11, enhance the tissue factor pathway.

## 3. Coagulation Factors and Their Antibacterial Role during Sepsis

Factor VII (FVII), FIX, and FX are able to initiate a coagulation cascade via their serine protease activity [5]. Nevertheless, the coagulation factor deficiency seems to have a significant correlation with bacterial infectious diseases, and it was proposed that these coagulation factors may have anti-infection properties [6,7]. In a study of Chen et al. [8], the authors reveal their potent antibacterial activity against Gram-negative bacteria, proposing a concept that FVII, FIX, and FX constitute a class of important antimicrobial proteins.

## 4. Toxins

Since the discovery of bacteria being able to produce toxins, such as the Shiga toxin, a clear clinical association with thrombotic and hemorrhagic clinical complications has been found. Toxins, such as Shiga toxin or Shiga-like toxins, have the ability to be produced by Gram-negative enterobacteria, such as *Shigella* spp., *Escherichia coli* spp., or others. From a pathophysiological point of view, the Shiga toxin is able to activate a complementary cascade and then the clotting intrinsic pathway via the activation of factor XII with the kinin system. Furthermore, due to endothelial damage, an increased release of clotting factor VII is able to activate the extrinsic pathway of the clotting cascade. Endothelial damage also induces the release of P-selectin and induces the activation of leukocytes and platelets, amplifying related dysfunctions due to cytokine release. Thrombosis is the final result, and it appears in small vessels, most commonly in the kidney. For this reason, hemolytic uremic syndrome is a common clinical complication of this toxemia. However, other thrombotic microangiopathies may appear regarding thrombotic thrombocytopenic purpura. Finally, high levels of serum ICAM-1 are associated with the development of MOF. Moreover, high levels of VCAM-1 are associated with both MOF and in-hospital mortality [9].

## 5. Endotoxin

The endotoxin lipopolysaccharide (LPS) found in the outer membrane of Gram-negative bacteria is a strong trigger of septic immune complications and of coagulation abnormalities during sepsis. LPS is a toxic molecule complex, composed of three distinct domains: lipid A, which possess most of the biological properties of LPS; the R core oligosaccharide; and the highly variable O antigenic polysaccharide. Due to its antigenic domains, LPS is able to interact with clotting factor VII, inducing its activation. After its activation, factor VIIa is able to induce the coagulation cascade via the activation of zymogen of clotting factor X. Therefore, factor Xa changes prothrombin into thrombin, leading to the generation of fibrin. Furthermore, LPS may also interact with cellules or their active fragments, such as platelets. The expression of the LPS receptor named “TLR4” on the surface of platelets, in fact, confirmed it. After interacting with TLR4, platelets are able to express their procoagulant activity through an interaction with coagulation factors, fibrinolytic factors, and endothelial products.

## 6. Porins

Aquaporins (AQPs) are a family of passive integral membrane proteins that facilitate the highly efficient yet strictly selective passage of water and small solutes across cell membranes [10,11]. The first AQP discovered was human AQP1 of the subfamily of pure water channels [12,13]. AQPs form tetramers to function as water pores. Data from AQP-knockout mice and from humans with loss-of-function mutations in AQPs demonstrate their role in epithelial fluid secretion, cell migration, edema, and procoagulant activities, especially during sepsis and bacterial infection. This suggests that the modulation of AQP expression or function may have broad clinical indications, such as in the treatment of glaucoma, intracranial hypertension, cancer, obesity, brain injury, and thrombosis [14]. On the other hand, porins may act as toxins, damaging different systems and organs in the host. For this reason, they play a fundamental role in the processes involved in drug resistance and bacterial-induced complications, such as thrombosis, bleeding, and septic shock. In particular, in vitro studies in the early 2000s showed that bacterial porins enhance thrombin activity upon interacting with the chromozym, a chromogen substrate [15]. This study suggests that bacterial porins might contribute to an increase in the risk of thrombosis during several illnesses, especially during sepsis and bacterial sepsis. Two molecules were identified as the possible targets of the procoagulant effect of porins: thrombin and fibrinogen, or both. It was demonstrated that Gram-negative bacteria porins strongly enhance fibrin formation because of thrombin hyperactivation and a reduction in AT-III activity. Bacterial porin from salmonella is able to activate the clotting cascade with the direct activation of thrombin [16], explaining its frequent association with thrombosis and disseminated intravascular coagulation (DIC). Several classes of porins (MW: 35.000 Da) are embedded in the cell membranes of Gram-negative bacteria, which control cell permeability by forming cross-membrane channels [17,18]. Together with porins, Gram-negative bacteria also contain lipopolysaccharide (LPS) [18]. LPS is able to induce tissue factor formation from monocytes or endothelial cells [19]. This occurs via the activation of circulating clotting factor VII, thus resulting in enhanced coagulation. It was found that micromolar concentrations of porins from salmonella markedly accelerated human blood coagulation in vitro. The main target of the porin-induced procoagulant effect was thrombin, without an increase in the activation of factor VII. A possible binding of porins with thrombin has been suggested to be the basis of this effect [20]. Because of these actions in vitro, these reported mechanisms could be at the basis of the association of the clinical features among septic shock due systemic salmonella infections and disseminated intravascular coagulation (DIC).

## 7. Association among Sepsis and VTE

Sepsis is a life-threatening syndrome that can rapidly evolve into multiorgan failure due to a dysregulated endocrine, immune, and metabolic response to infection [20]. Septic patients are generally affected by coagulation disorders [21]. For these reasons, septic and multiorgan failure septic patients are at a high risk of thrombotic complications, including localized and diffused microvascular involvement, disseminated intravascular coagulation (DIC), and venous thromboembolism, which includes deep vein thrombosis (DVT) and pulmonary embolism (PE) [22,23]. Virchow’s triad represents, historically, the basis of the pathophysiological processes leading to thrombosis that are characterized by (1) vascular wall damage or dysfunction; (2) blood flow alterations; and (3) hypercoagulability [24,25]. However, Virchow’s triad alone is no longer sufficient to explain the complex mechanisms that produce thrombotic events during hospitalization for sepsis. A growing body of evidence has suggested the role of inflammation in the pathophysiology of VTE during sepsis [25], highlighting a correlation between venous thrombosis and inflammatory disorders, adding contextual elements to the classical Virchow’s triad. During sepsis, several pathophysiological processes occur simultaneously [21], and among them, the dynamic relationship between coagulation and inflammation assumes much more relevance. The platelet–leukocyte interaction through P-selectin and P-selectin glycoprotein ligand 1 (PSGL-1) [26] and the stimulation of platelets through Toll-like receptor 2 (TLR2) [27] are two of the most important underling mechanisms. In 2013, the term “immunothrombosis” was coined by Engelmann and Massberg [28] to explain the complex and reciprocal interaction between the immune system and bacterial pathogens. The activation of a coagulation cascade triggers the immune system, cooperating with the identification, containment, and destruction of pathogens [28]; on the other hand, innate immune cells promote the development of thrombus [22]. In this context, many actors are involved, starting from the endothelium, platelets, coagulation factors, cytokines, immune cells, and their interactions with bacterial toxins, which are specific products of bacteria able to activate a clotting cascade with an induced infective hypercoagulable state. Some bacteria are capable of producing cellular membrane proteins, such as porins, which can mediate the complex prothrombotic mechanism present in patients with septic manifestations. This appears to be at the basis of deep vein thrombosis and microvascular phenomena [29,30]. Interestingly, despite a deeper understanding of the pathophysiology underlying VTE in sepsis and the strict relationship between these two conditions, the current therapy available for VTE consists of prophylactic or therapeutic strategies targeting coagulation factors without considering the inflammatory processes that lead to thrombosis, leaving a significant gap in determining the effective treatments for and the prevention of VTE.

## 8. Association among Infection and DIC

Progressive multiorgan failure is common in patients with sepsis or septic shock and is usually due to the overlapping of DIC. Disseminated intravascular coagulation (DIC; also called consumption coagulopathy and defibrination syndrome) is a systemic process with the potential to cause thrombosis and hemorrhage. It can present as an acute, life-threatening emergency or a chronic, subclinical process, depending on the degree and tempo of the process and the contribution of morbidities from the underlying cause. Identifying DIC and the underlying condition responsible for it are critical for proper management. For these reasons, active infections are considered as a major risk factor for DIC. Several recombinant drugs with clotting effects, such as antithrombin or protein C, have been tested in dedicated studies besides the use of heparins and fresh frozen plasma.

A recent review on the efficacy of recombinant antithrombin in this clinical setting underlined an improvement of DIC evolution in treated patients, although analyzed data from pooled randomized clinical trials did not show improvements in survival rates.

Nonetheless, a reduction in mortality in this clinical setting has been reported by a combined recombinant treatment based on the administration of recombinant antithrombin and recombinant thrombomodulin [31].

Based on the data available since the publication of the PROWESS-SHOCK trial, the use of recombinant protein C is not suggested by international guidelines because the improvements of laboratory data are not associated with improved outcomes of treated patients. Moreover, an increased rate of bleeding has also been reported in treated patients, both in children and adults. Therefore, the current evidence does not support the use of human recombinant activated protein C in adults or children with septic shock, and an associated risk of bleeding needs to be considered when this drug is used for any reason [32]. The benefit of the early administration of fresh frozen plasma (FFP) has been frequently tested in patients with septic shock; however, the inappropriate administration of increased doses of FFP is associated with poor outcomes for treated patients. Therefore, FFP remains a good choice for treating DIC in septic patients but to reduce the inappropriate use of FFP. Furthermore, avoiding unnecessary adverse transfusion reactions is one of the next golden standards to be raised in the coming years [33].

Intriguingly, the early administration of low-molecular-weight heparins in patients with septic shock can improve not only laboratory data; it is also associated with a reduction in inflammatory biomarkers and clinical outcomes and survival of treated patients, particularly regarding multiorgan dysfunctions and multiorgan failures [34]. This clinical benefit may mainly be due to the therapeutic anti-inflammatory effect of heparins, apart from their anticoagulant effects [35].

## 9. Association among Sepsis and TMA

There are several mechanisms by which *Escherichia coli* spp. is able to release Shiga toxins and induce a procoagulant state.

Firstly, these kinds of infections induce enteritis with inflammatory local damage, which has the potential to induce systemic inflammation. Systemic inflammation leads to the release of cytokines that mediate damage caused to the kidney, resulting in the onset of the hemolytic uremic syndrome. The activation of systemic inflammation and the increased release of cytokines induces changes in endothelial functions. Secondly, activated protein C is able to remove factor Va and factor VIIIa via selective cleavage. The activation of protein C is mediated by thrombin, but thrombin inhibition is increased during inflammation because of the increased release of thrombomodulin due to endothelial dysfunctions. For this reason, the procoagulant state during *E. coli* spp. infections and the subsequent release of the Shiga toxin is associated with small-vessel thrombosis and thrombotic microangiopathies. Thirdly, increased thrombin generation during infections/inflammations is also associated with an increased release of thrombin activatable fibrinolysis inhibitor (TAFI) [36]. It inhibits fibrinolysis by preventing the conversion of plasminogen to plasmin, an enzyme responsible for clot dissolution. The combined action of increased levels of TAFI and the plasminogen type 1 inhibitor are, in fact, able to slow down thrombus resolution, enhancing the thrombotic power in thrombotic microangiopathies.

## 10. Plasma Exchange and Toxins

The damage induced by toxins may be modulated by the clearance induced by plasma exchange. Hemodialysis and hemofiltration can remove high-molecular-weight proteins or protein-bound molecules (i.e., >than 40 kDa), including bacterial toxins and porins [37,38]. Filters used to perform plasmapheresis are also able to remove high-weight proteins up to 60 kDa, including bacterial porins. For this reason, during HUS, hemodialysis, hemofiltration, and/or plasmapheresis remain a golden standard treatment when seeking improvements in kidney failure and in toxin/porin removal.

Of course, these types of treatments should be associated with a causal diagnosis of sepsis and be used as a specific antimicrobial treatment for stopping toxin\porin production caused by bacteria.

## 11. Conclusions

Several mechanisms induced by bacterial toxins and porins are able to induce an acquired hypercoagulable state during sepsis. Despite this, some of these mechanisms reported in vitro and in vivo may be responsible for fatal clotting complications found in septic patients, such as in patients with disseminated intravascular coagulation or thrombotic microangiopathies with multiple organ failures. One of the next objectives of clinical research may be to focus on porin and toxin inhibition with the goal of tackling thrombotic complications.

## Figures and Tables

**Table 1:** Predominant bacteria involved during sepsis.

Bacteria Involved in Sepsis	
**Gram-positive bacteria**	Methicillin-sensitive *Staphylococcus aureus* (MSSA), methicillin-resistant *Staphylococcus aureus* (MRSA), *Staphylococcus epidermidis*, *Staphylococcus* spp., *Streptococcus pneumoniae*, *Streptococcus pyogenes*, *Streptococcus agalactiae*, *Streptococcus dysgalactiae* spp. equisimilis (SDSE), *Streptococcus anginosus*, *Streptococcus constellatus*, *Streptococcus* spp., *Enterococcus faecalis*, *Enterococcus faecium*, *Enterococcus* spp., other gram-positive cocci, *Clostridium difficile*, *Clostridium perfringens*, *Clostridium tetani*, *Clostridium* spp., other gram-positive rods
**Gram-negative bacteria**	*Moraxella catarrhalis*, *Neisseria meningitidis*, *Neisseria* spp., *Acinetobacter baumannii*, *Acinetobacter* spp., *Aeromonas hydrophila*, *Aeromonas* spp., *Bacteroides fragilis*, *Bacteroides* spp., *Burkholderia cepacia*, *Burkholderia* spp., *Citrobacter freundii*, *Citrobacter* spp., *Escherichia coli*, *Enterobacter* spp., *Haemophilus influenzae*, *Klebsiella pneumoniae*, *Klebsiella oxytoca*, *Klebsiella* spp., *Legionella pneumophila*, *Legionella* spp., *Pseudomonas aeruginosa*, *Pseudomonas* spp., *Proteus mirabilis*, *Proteus* spp., *Rickettsia* spp., *Salmonella enteritidis*, *Salmonella* spp., *Serratia marcescens*, *Serratia* spp., *Stenotrophomonas maltophilia*, *Stenotrophomonas* spp., *Vibrio vulnificus*, *Vibrio cholerae*, *Vibrio* spp., gram-negative rods

## Data Availability

No new data were created or analyzed in this study. Data sharing is not applicable to this article.
